# Double Threshold Prioritarianism – Some Problems and Solutions

**DOI:** 10.1007/s10728-025-00517-2

**Published:** 2025-03-28

**Authors:** Adam Ehlert

**Affiliations:** Centre for Research Ethics and Bioethics (CRB), Uppsala Universitet, Uppsala, Sweden

**Keywords:** Distributive justice, Health care, Prioritarianism, Priority setting, Sufficientarianism

## Introduction

In any healthcare system, there is a need for distribution of resources. Healthcare resources are scarce, and we must decide where to spend them. One way of doing this is to agree on a set of morally sound principles for distribution of healthcare resources. These principles should guide us in choosing whom to treat first, or, sometimes, at all (which treatments to subsidize, which condition we should prioritize, which condition-treatment couples we should prioritize, etc.). This is, very roughly, what is referred to as priority setting in health care.^1^

One kind of normative principle sometimes used in priority setting is some principle of *severity*; which roughly states that we should prioritize based on severity of illness. Basically, the more severe a condition, the higher the priority given to that condition (all else being equal).^2^ There is broad public support for the idea that more of scarce resources should be distributed to more severely ill patients [1, 2]. In, for instance, Sweden, Norway, the Netherlands, and most recently the UK [3–6], severity has been explicitly adopted as a priority-setting criterion by accepting a higher cost per health benefit the more severe a condition is [7]. Despite all this, severity is still an under-theorized and contested concept [8, 9].

One issue regarding severity that requires further clarification and discussion is how severity should be understood in relation to interpersonal aggregation [8, 10]. When we do moral (or economic) calculus, in what way (if any) are we allowed to “add up” and compare different “severities” (that is, different groups of people with certain conditions)? If we accept the common intuition behind the severity principle that it matters more the more severe a condition is, it can seem quite contrary to such an intuition to claim that a larger aggregated benefit for people that are better off (i.e. with lower severity) should outweigh a smaller aggregated benefit for the worse off (i.e. with higher severity). The purpose of this paper is to discuss a proposed a solution to this so-called *aggregation problem*.

*The aggregation problem*. From a severity perspective, there are some types of aggregation that seems reasonable, while others seem unreasonable.

Gustavsson and Juth [11] has suggested a principle, supposed to escape this problem, that they call Double Threshold Prioritarianism (DTP). DTP allegedly solves the aggregation problem by combining a prioritarian framework with elements of sufficientarianism. While the sketch they present is interesting, I believe it needs further development and clarification. In this paper, I will take my point of departure from the distributive principle of prioritarianism. I will briefly describe prioritarianism, as well as the reasoning behind DTP. I will then make some clarifications and developments of the original theory. In their original article, the authors also present some problems that their principle could encounter. I will therefore also discuss some of these problems and explain how they problems can be avoided, further strengthening the theory. But first, I will make some general clarifications, before providing a more thorough introduction to the aggregation problem,

## Clarifications

In this paper, I will speak of ”health” as the general currency that severity is supposed to relate to. This is to be very roughly understood as health-related quality of life, that is, the parts of a persons well-being that is relevant to health. It is debatable whether it is possible to separate health from general well-being [12], but for this paper, this discussion will be bracketed.^3^ It will simply suffice to note that the more health one has, the better off one is, and the less, the worse off one is (all else being equal). Again, this is only for simplicity, and I do not make any claim that it is the most suitable way to understand worse-offness. I will assume that we can rank people with regards to ”health” on a scale from 0 to 10, where 0 is worst possible health and 10 is best possible health. Correspondingly, 0 constitutes highest possible severity, and 10 lowest possible severity. I also assume that every unit on this scale is of equal size and that we can fit every health care need on this scale.^4^ Again, this is a gross simplification that is useful for our theoretical purposes.

I will also remain neutral on the temporal scope of the assessment. There are in practice three different ways to consider the relationship between time and worse-offness. Either we consider the entire life,^5^ or we consider only the present moment, or we consider some specific segment of the life (like the future or the past^6^). My argument will not presuppose commitment to any of these views.^7^ Hence, the purpose of this paper is not present any arguments against any of these views and principles, but only to present a positive argument for DTP.

There has been extensive philosophical debate regarding moral aggregation in recent years. In his 1997 paper, Norcross [17, 18] points out what I call *the aggregation problem*, using the *Headache* example.^8^ While Norcross argues that the problem is unavoidable and that at least consequentialists should embrace it (what I call UIA), many other writers have disagreed [19–25]. It is a common view shared by many contemporary philosophers that aggregations of the *Headache* kind are wrong and that aggregations of the *Fracture* kind are not wrong. (It is, of course, not shared by everyone [26–28]). To defend this, one has to provide some sort of principle, such as Voorhoeve’s ”Aggregate Relevant Claims” principle [29]. While such principles can encounter problems of their own [30, 31], I will not go further into this discussion, because it is an important point to make that the general discussion is centered on the theoretical issue: whether aggregation is possible and/or if it should be accepted (by consequentialists). In this paper, the question is about what types of aggregation the health care system should allow out of respect for illness severity. My point in this paper is not to engage in the theoretical discussion of aggregation, and the reason for including these cases in this paper is to set the scene for the discussion. I will therefore set the more theoretical discussion aside, while pointing out that even an unrestricted view such as Norcross’s [17] could be compatible with the framework discussed in this article:^9^ a limited aggregation-based *principle for severity-based priority setting c*ould be compatible with a fully unrestricted view of aggregation *simpliciter*.

In the next section, I will give a brief introduction to the aggregation problem, before moving on to prioritarianism and DTP.

## The Aggregation Problem

Consider this example:

*Headaches*.

10

We have to choose between either curing one person of a life-threatening type of pancreatic cancer or curing 40 million people each of a 30-minute headache. Since the aggregated severity of the people with the headaches is, it turns out, strictly bigger, than that of the one person with the cancer, we ought to cure the headaches.^11^

Many people would find this wrong. It seems especially wrong from the point of view of severity. “No amount”, they say, “of minor headaches cured can outweigh curing (saving the life of) someone with cancer”. A severity principle seems, based on this, directly in opposition to what we could call *unrestricted interpersonal aggregation (UIA)*. As the name implies, UIA allows for any and all types of aggregation. Judging by intuitions from cases like *Headache*, it seems as though we would want to forbid such aggregation. But consider this second example.

*Fractures*.

We have to choose between curing one person with a fractured leg or curing two people with a fractured arm. Since the aggregated severity of the persons with the fractured arms is, it turns out, strictly bigger, than that of the one person with the fractured leg, we ought to cure the fractured arms.^12^

This does not seem obviously wrong. (If you do find it wrong, it should be possible to substitute the conditions for some other pair of conditions.) We agree that it is worse to break one’s leg than one’s arm. Maybe this is the case because a broken leg adversely affects one’s daily life to a higher degree. However, it does not seem *unreasonable* to choose to cure two broken arms instead of one broken bone. This seems to suggest that even from the point of view of severity, we should allow *some* types of aggregation of health benefits. We simply cannot rule out all aggregation on the basis of *Headache*-like cases. There seem to be (based on the above cases) some types of aggregation we want to allow, but we do not want to allow unlimited aggregation. We might say that the line should be “somewhere in the middle”. But where?

It is also possible to pose the question in a different way, namely – which groups should be allowed to benefit at the expense of which other groups? When we do priority setting, we move health care resources between different patient groups. Any time we devote resources to a specific group, this constitutes an opportunity cost for some other groups, since the money could have been spent elsewhere. When are we justified in doing so? Here, a need for a more specific distributive principle enters, in order to give an answer to how we should distribute health care resources.

## Prioritarianism

Prioritarianism [32] is a principle of distributive justice whose underlying reasoning closely resembles that of the severity principle. Prioritarian theories suggest that we should give explicit priority to those who are worse off. We do that by holding that a given benefit matters more the worse off the recipient is. As an axiological principle, used when comparing different states of affairs, where w_n_ denotes the well-being of a specific person, prioritarianism can be formulated:

A state of affairs x = (w1, w2,…, wn) is at least as good as another state of affairs y = (w1’, w2’,… wn’).

if and only if.

[*f*(w_1_) + *f*(w_2_)+…+*f*(w_n_) ]≥ [f(w_1_’) + *f*(w_2_’)+…+*f*(w_n_’)], where *f*( ) is some strictly increasing, strictly concave function [10]. 

One suggested value for *f* is the square root [10]. This gives us a principle on which the well-being of someone better off counts relatively less than that of someone worse off. A given benefit is therefore better given to someone worse off, since prioritarianism is in one regard a maximizing principle. There seem to be several reasons to favor prioritarianism over, for instance, egalitarianism—in health care priority setting, especially from the point of view of severity [33].

However, it must be noted that prioritarianism as formulated above can allow for unlimited interpersonal aggregation. A significantly large aggregated benefit to people with high health (hence, low severity) can outweigh a benefit to someone with lower health (and higher severity). If we go back to the headache case, more headaches would be required to outweigh a death. As such, prioritarianism only *tempers* the aggregation problem. Therefore, it is still the case that the way it deals (or, rather, doesn’t) with aggregation makes it problematic from the point of view of severity.

One way of tempering this problem with prioritarianism could be to combine it with some elements of sufficientarianism. Sufficientarian justice, very briefly, aims at making sure that everyone has “enough”, that is, there is some sort of threshold that we should make sure that *everyone* or *as many people as possible* are above [22, 34]. Gustavsson & Juth [11] have proposed such a theory, that they call “Double Threshold Prioritarianism” (DTP). DTP, allegedly, allows us to keep the promising parts of regular prioritarianism, while providing a solution to the aggregation problem.

## Double Threshold Prioritarianism

If we consider our ranking of people’s health from a scale of 0 to 10, prioritarianism says that it matters more to benefit a person the worse off they are, so if we are able to raise *one* person with 1 unit, we should give that unit to the one person that is lowest on the scale. (A strictly utilitarian principle, for instance, would be indifferent as to who gets the unit.)

Gustavsson & Juth first apply “regular” prioritarianism (perhaps with the square root function) all across the population, from 0 to 10. They then add two thresholds. The exact level of these thresholds is, they say, subject to discussion, but they put them at 2 and 8. Now, the population is divided into three groups, which they call, aptly, “the worst off”, “the moderately badly off” and “the best off”. What the thresholds are *doing* here is *prohibiting* the redistribution of health benefits between groups according to certain rules. In particular, they forbid redistribution from “the worst off” to “the best off”. Even if the *aggregated* benefit (with regards to severity) for some people among the best off would be strictly bigger than that for the worst off (such as in *Headache*), on DTP, such redistribution is forbidden. Gustavsson & Juth asks us to consider the following cases. If we have to choose between (a) providing a minor benefit to an individual among the worst off or (b) providing a large benefit to a large enough number of (say a hundred thousand) people among the moderately badly off, we ought to choose (b). If we have to choose between (b) and (c) providing even larger benefits to a billion people among the best off, *and we cannot choose (a)*, we ought to choose (c). The important matter is that if we have to choose between (c) and (a), *and we cannot choose (b)*, we *always* ought to choose (a). This is the work the thresholds do – they forbid direct redistribution from the worst off to the best off.

DTP is intuitively attractive, primarily because it seems to provide us with a way to keep the prioritarian theories while solving the aggregation problem. It gives the intuitively right verdict in cases such as *Headache* and *Fractures*. Let us for the sake of argument grant that the people with headaches are among the best off, and the cancer patient among the worst off. Let us also grant that the patient with a fractured bone *and* the patients with fractured arms are among the moderately badly off. If we stipulate this, DTP gives acceptable answers in all cases.^13^

DTP can also avoid a common critique against standard uni-threshold sufficientarian theories because it tells us *how* to prioritize among people that are below (or above) the threshold(s): in each group, a prioritarian function applies. We also give *absolute* priority to “the worst off” over “the best off”, but this relation does not hold between any other combination of groups.

DTP has good potential at tempering some problems with prioritarianism in healthcare and can as such be useful from the point of view of severity. However, the theory needs further discussion and elaboration to be a viable contender for a distributive principle. In the following section, I will provide some further development of the theory, after which I will move on to discuss its potential shortcomings.

## Further Development of DTP

One important aspect of DTP that Gustavsson & Juth do not explicitly point out is that, since the thresholds entail prohibitions, they are, so to say, *deontological* thresholds. The work these thresholds are doing in a theory such as DTP is different than the work thresholds do in the sufficientarian theories proposed by, for instance, Crisp [22]. In those theories, it is very important to move people to or above a threshold, which makes the threshold carry a substantial moral weight. With DTP, it is not in itself important to move people over the thresholds, and nor is it concerned with any sort of maximizing of the amount of people over a certain threshold. It might therefore be more sensible to instead consider the thresholds *constraints*. It is then more clear that we end up with a combination of a distributive principle with some absolute rules that forbid (certain) tradeoffs. It should, again, be pointed out that as DTP deals with resource distribution (in the health care setting), it makes claims on when and to which groups we are allowed to (re)distribute resources.

**Fig. 1:**
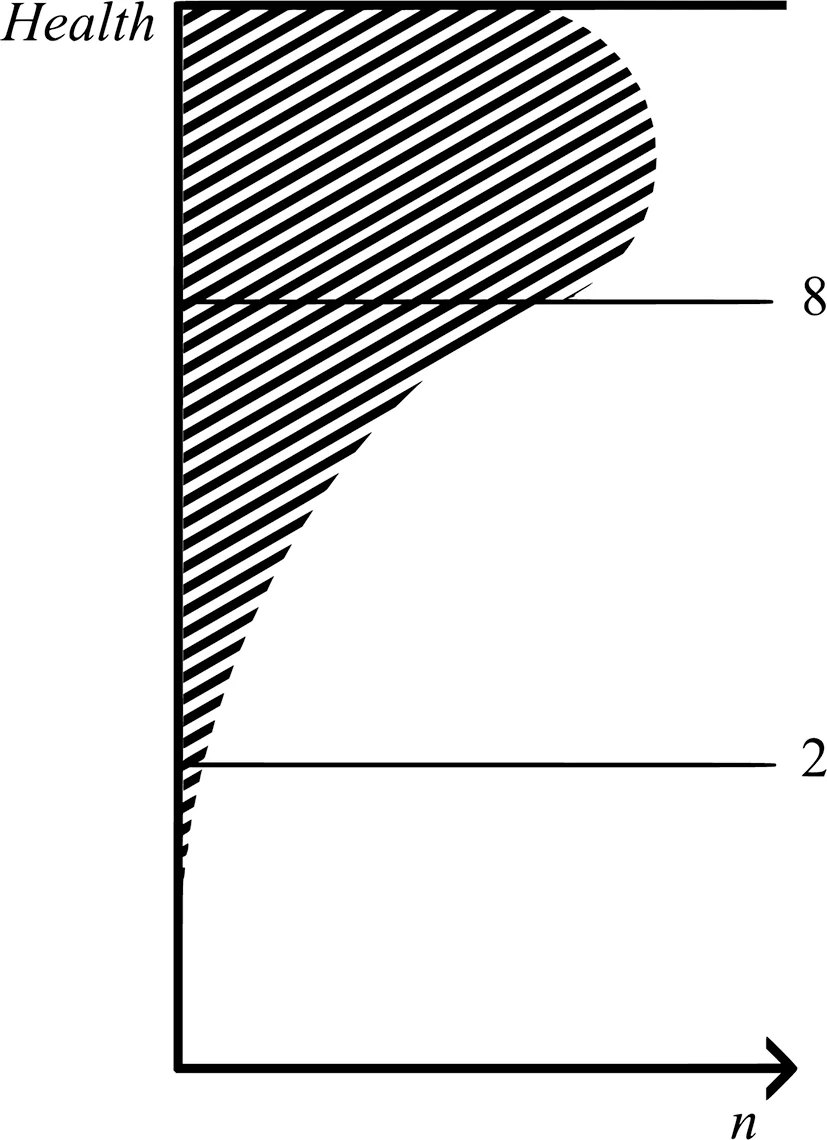
A distribution of health with thresholds at 2 and 8

It can be helpful to see a sketch on such a distribution (Fig. 1). Here, the x-axis represents health (and, via simplification, severity), and the y-axis represent the number of individuals positioned at the given level. It is reasonable to assume that the amount of people among the best off is bigger. The different fields correspond to the different groups (a), (b) and (c) that DTP stipulate. The horizontal lines illustrate the two ”thresholds” (as well as the stipulated bottom and top level). Looking at this figure, it is perhaps easier to understand the underpinnings of DTP.

Even with these clarifications in mind, DTP might still seem problematic. It is first reasonable to ask how it is supposed to evaluate cases. Is the evaluation done *ex post* or *ex ante*?^14^ Imagine that we have two people, Jack and Jill, with respective health levels of 9 and 2. Both Jack and Jill acquire some type of condition that, if untreated, will result in their health level being 1. Imagine that we have to choose one of them to treat. (If treated, we assume they return to their respective previous levels of health.) Then, it might seem that DTP says that we should treat Jill, that goes from 2 to 1 (resulting in 1 unit of health), and not Jack, that goes from 9 to 1 (which would result in 8 units of health). This might seem like the wrong thing to do.

Considering this, *ex ante* evaluations of the case will point in favor of treating Jill. Coupled with the deontological constraints of DTP, this will be the option chosen. The problem here is that severity is conflated with *capacity to benefit*. The reason why it might (to some) seem wrong to treat Jill is that we can benefit Jack more — we are able to bring him up to almost full health, and restore 8 units of health. If we were to treat Jill, we would only restore 1 unit of health. What is important to stress here is that if the factor of *benefit* is held fixed, and we only focus on severity, it seems like the right thing to do. If the benefit is held fixed, it is the level of severity that will determine priority, and hence, Jill should be treated.

An *ex post* evaluation on the other hand, would consider Jack and Jill on an equal level of severity. They are both on health level 1, which is a high level of severity, and a severity principle would give no verdict on which one to treat. Here, capacity to benefit becomes important. If we then assume, with the case, that we can bring Jack up to level 9, and Jill only to 2, it seems reasonable to treat Jack — but this is not through work from some *severity* principle. This is some *other* principle doing work.

It might be reasonable to think of DTP as a time-neutral theory, in that it is not committed to make either *ex ante* or *ex post* judgements, but instead refers to an idea of severity that is *sub specie aeternitatis*. DTP is not sensitive to which level of health (or severity) people *used* to have. It is a mid-level with the purpose of putting up certain deontological rules to temper the aggregation problem of prioritarianism. It has to be applied together with other mid-level principles. Because of this, DTP would be neutral in the case just presented.

DTP might have some other problems. The original authors of DTP briefly discuss some potential objections. In the following, I will describe and expand upon these objections, that I call *Arbitrariness* and *Intransitivity*, and I will defend the theory against them.

## Arbitrariness

It might be opposed that the placement of the thresholds is arbitrary. What reason do we have to put them at level 2 and 8, or any other level? Especially for an individual just under a threshold, this seems like a strong complaint.

This objection has been posed against sufficientarianism [22, 34], and against especially headcount sufficientarianism, they have some force [35, 36]. In certain sufficientarian theories, the threshold itself has (big) moral importance. There exists in such theories an imperative to move people over or up toward the threshold. Theories vary in their exact formulation, but perhaps we want to get as many people above the threshold as possible, get as many people as possible below the threshold as close to the threshold, or some variation. For a sufficientarian theory to escape this objection, the placement of the threshold has to be well-grounded in theoretically sound reasoning. It is therefore important to be careful when placing the thresholds. A reasonable starting point might be to divide conditions into four different groups (very severe, severe, moderately severe, and not severe) and attach the thresholds at the higher end of the first group and the lower end of the last group. Such a reasoning could be based on, for instance, absolute QALY shortfall [4].

But, DTP is not a sufficientarian theory — it is again important to de-emphasize the ”sufficientarian element” of the theory and instead shift to talk about constraints. DTP has its basis in a prioritarian theory. Such theories say that a given benefit matters more the worse off the person is that is given that benefit. As such, there are strong reasons to make life better for people who are worse off, but not in relation to any sufficientarian ”threshold”. If we move to think about the thresholds as deontological constraints, it becomes easier to understand the type of work they’re doing; prohibiting redistribution between certain groups, as described above. The most important aspect of DTP is the (theoretical) distance that the constraints create between the worst off and the best off, the two groups between which redistribution is forbidden (in the bottom-top direction). A first proposal to avoid the arbitrariness criticism is to claim that the exact position of this distance might not be of utmost importance, but might instead depend on the given distribution. Again, it is important to stress that the aim of DTP is to give priority to the worst off while forbidding certain types of aggregation, which results in giving *absolute* priority to the very worst off. The importance is not on moving people over thresholds.

The exact *number* of constraints is another important aspect. Gustavsson & Juth chose to use two thresholds, and I have translated that into what is essentially one constraint. It is not necessary to add more thresholds for the theory to do the work that it is supposed to do (prohibiting unrestricted aggregation while allowing some types of aggregation). An interesting aspect of this is that if one wants to be more specific regarding which types of aggregation one wants to forbid or allow, it is possible to input additional thresholds at what are deemed to be appropriate levels. However, on the original formulation of DTP, the two thresholds can be translated into one constraints, that forbid redistribution from the worst off to the best off.

## Intransitivity

Another criticism that can be raised against DTP (and other multi-threshold theories) is that it violated the (alleged) transitivity of the betterness relation. The betterness relation (A is better than B) is, by many philosophers, said to be *transitive*.^15^ DTP might seem to suggest that A can be better than B, B better than C, and C better than A. Therefore, the objection goes, the theory violates transitivity, and therefore ought to be rejected.

There is an extensive debate whether the betterness relation has to be transitive or not [37–45]. While it is certainly possible to argue, in accordance with, for instance, Temkin [38, 39], that the betterness relation does not have to be transitive, there is an easier way for adherents of DTP to meet this objection. Simply put, the groups described in DTP do not stand in a betterness relation to one another, but some more complex relation. We might think of the this relation as ”ought to be given priority (under condition x, y, z…)” or something similar. The reason for claiming that betterness demands transitivity is that it is (in certain aspects) a *simple* type of relation. The relation just describe, or whichever relation the groups in DTP stand toward each other in, is not such a simple relation. It is a relation that holds under certain conditions.^16^

Moreover, real-life priority setting is poorly modeled by any social welfare function requiring transitivity. The choices actually made in practice are often ”intransitive”, as we can see when using incremental cost-effectiveness analysis or multi-criteria decision making tools. With these comments in mind, I believe that DTP can avoid the transitivity objection.

## Conclusion

In this article, I have presented and expanded upon a distributive principle called “Double Threshold Prioritarianism” (DTP). I have argued that this principle is reasonable from the point of view of severity, since it is prioritarian while at the same time restricting unlimited interpersonal aggregation. I have clarified that DTP is a mid-level principle of distributive justice that makes use of deontological constraints to prohibit certain tradeoffs. I have then defended this principle against two possible objections – the arbitrariness objection and the transitivity objection.

It is a possible implication of DTP that there are very little (or none at all) resources left to be distributed to the people among the best off. Some may consider this worrisome. Here, it is important to bear two things. The first is that DTP is not made to be the *only* principle used in a priority setting platform — the results of DTP have to be balanced with other principles, such as need and cost-effectiveness. The second is that health care priority setting and theories such as DTP assume that we are working under conditions of (moderate to severe) resource scarcity. There is, simply put, not enough cake for everyone to share. Under such circumstances, it is good if the resources are distributed with sensitivity to severity: more resources should be given to people who are worse off. But I am prepared to go a step further and assert that it is (instrumentally) good that (again, under conditions of scarcity) the best off are not given resources. Under conditions of abundance, I would be happy to revert to some fully aggregative principle and simply give everyone as much as possible. But since we are dealing with considerations of scarcity, it is good that resources are not given to the people over the upper threshold. That is so because that ensures that they can be spent on people who are worse off.

## Footnotes

1 Henceforth ”Priority setting” (for brevity).

2 In practice, the severity principle is often used as a modifier for a principle of cost-effectiveness; that is, the higher the severity, the higher the threshold for what is seen as cost-effective. We are simply willing to pay more per health benefit for the treatment of more severe conditions.

3 The reader could substitute for health other measurements such as Health-Related Quality of Life, Quality-Adjusted Life Years or general well-being. All that is being claimed here is that the currency is measurable on some sort of discrete scale.

4 It is very reasonable to suspect that there could be people below the zero-level, that is, people with health states worse than death. This is of interest to any axiology but I will simply set that aside. For our purposes, we can simply imagine that 0 is the worst possible level of health.

5 Such as Lifetime QALY Prioritarianism [13] and the Complete Lives Framework [14].

6 See Herlitz 2018 [15].

7 For a detailed discussion of the relation between time and illness severity, see Sandman & Juth [16].

8 Scanlon discusses the same case, as well as a similar example, called ”The World Cup Case”, to illustrate what I consider basically the same point [21], Since this paper discusses health care priority setting, I find it more fitting to use the ”Headaches versus lives” case from Norcross and others.

9 For two good summaries of this debate, see Hirose [10] and Horton [31].

10 This is a version of a case originally discussed by, among others, Norcross [17] and Scanlon [21].

11 It is assumed here that life-threatening pancreatic cancer is very severe, and that headaches are not. We also assume here that we are “saving the life” of the cancer patient. While this is not strictly possible, since we can only prolong it, assume that if we do not cure the cancer patient, she will die almost immediately. If we do cure the cancer patient, she will return to full health and live until, say, 80. If we do not cure the headaches, these people will have a 30-minute headache and then return to full health and live until 80. If we do cure the headaches, these people will return to full health and live until 80.

12 As in the previous example, we assume that a broken bone is more severe than a broken arm. Also, as in the previous example, if cured, the people are returned to full health.

13 It should be noted that even if the people in *Fractures* are on either side of a threshold, DTP still gives the acceptable answer and allows the redistribution. It only forbids it if the patients have two thresholds between them – that is, if they are separated by the “middle block” of moderately badly off people.

14 Thanks to Greg Bognar for stressing this point to me.

15 If A > B, and B > C, then A > C.

16 If P, x > y, if Q, y < x.
